# Transcriptome profiling of genes involved in induced systemic salt tolerance conferred by *Bacillus amyloliquefaciens* FZB42 in *Arabidopsis thaliana*

**DOI:** 10.1038/s41598-017-11308-8

**Published:** 2017-09-13

**Authors:** Shaofang Liu, Haiting Hao, Xiang Lu, Xia Zhao, Yun Wang, Yubao Zhang, Zhongkui Xie, Ruoyu Wang

**Affiliations:** 1 Gaolan Station of Agricultural and Ecological Experiment, Northwest Institute of Eco-Environment and Resources, Chinese Academy of Sciences, Lanzhou, China; 2 Key Laboratory of Stress Physiology and Ecology in Cold and Arid Regions of Gansu Province, Lanzhou, China; 3 Key Laboratory of Desert and Desertification, Northwest Institute of Eco-Environment and Resources, Chinese Academy of Sciences, Lanzhou, China; 4 University of Chinese Academy of Sciences, Beijing, 100049 China

## Abstract

Plant growth-promoting *Bacillus amyloliquefaciens* FZB42 induces systemic salt tolerance in *Arabidopsis* and enhances the fresh and dry weight. However, the underlying molecular mechanism that allows plants to respond to FZB42 and exhibit salt tolerance is largely unknown. Therefore, we performed large-scale transcriptome sequencing of *Arabidopsis* shoot tissues grown under salt stress with or without FZB42 inoculation by using Illumina sequencing to identify the key genes and pathways with important roles during this interaction. In total, 1461 genes were differentially expressed (FZB42-inoculated versus non-inoculated samples) at 0 mM NaCl, of which 953 were upregulated and 508 downregulated, while 1288 genes were differentially expressed at 100 mM NaCl, of which 1024 were upregulated and 264 were downregulated. Transcripts associated with photosynthesis, auxin-related, SOS scavenging, Na^+^ translocation, and osmoprotectant synthesis, such as trehalose and proline, were differentially expressed by FZB42 inoculation, which reduced the susceptibility to salt and facilitated salt adaptation. Meanwhile, *etr1-3*, *eto1*, *jar1-1*, and *abi4-102* hormone-related mutants demonstrated that FZB42 might induce plant salt tolerance via activating plants ET/JA signaling but not ABA-dependent pathway. The results here characterize the plant transcriptome under salt stress with plant growth-promoting bacteria inoculation, thereby providing insights into the molecular mechanisms responsible for induced salt tolerance.

## Introduction

Soil salinity is a major issue that affects agriculture and approximately 20% of agricultural land is salt-stressed at present^1, 2^. Indeed, salt has become one of the main abiotic stress factors that limit agricultural productivity. Mechanistically, apart from causing plant ion imbalance and osmotic stress, excess salinity inhibits metabolism, including photosynthesis, protein and lipid synthesis, thereby limiting the crop growth and yield, and it may even lead to plant death^3, 4^.

In order to reduce the severe effects of salt stress on plants, many approaches have been developed. Many studies have addressed this issue by focusing on genetic engineering, but this approach is time-consuming and may cause possible environmental risks, so genetically engineered plants are rarely used in the field^5, 6^. Recently, the use of microbes has become a new alternative for improving stress tolerance in plants^7, 8^. Plant-growth-promoting rhizobacteria (PGPR) comprise a diverse group of rhizosphere-colonizing bacteria that promote plant growth via direct or indirect mechanisms^9, 10^, which may be correlated with the ability to resist various pathogens, the production of phytohormones, the release of volatiles, and the production of phytase and siderophores to enhance the availability of minerals in the soil^11–14^. In addition to their growth-promoting activity, some PGPR are also known to alleviate salt stress^15^ and the use of PGPR for salt tolerance enhancement has significant advantages compared with other approaches.

Many PGPRs have been studied and applied as useful and efficient agents for inducing salt tolerance in plants due to their ability to promote plant growth and to aggressively colonize plant roots. Recently, it has been shown that the tolerance of salt stress in many agricultural crops can be enhanced by various PGPR genera, such as *Azospirillum*, *Pseudomonas*, *Burkholderia*, *Bacillus*, *Arthrobacter*, *Azotobacter*, and *Enterobacter* ^16^. For example, inoculating plants such as wheat, radish, *Arabidopsis thaliana*, and maize with *Bacillus* sp. in saline conditions can prevent poor growth and improve the performance of plants in adverse conditions^17, 18^. The interaction between plants and PGPRs is a complex and reciprocal process. Thus, when PGPRs induce salt tolerance in plants, the expression patterns of plant genes will exhibit a corresponding response to their stimulation. However, most previous studies have focused mainly on the physiological aspects of plants during this interactive process and there have been no reports of the transcriptome profiling of genes involved in the induction of systemic salt tolerance conferred by PGPR in plants.

*Bacillus amyloliquefaciens* FZB42 was isolated from the rhizosphere soil of lettuce and it has been used widely in commercial applications to support the production of a broad range of economically important plants^19^. In recent years, many studies have been performed with FZB42 to determine its plant growth-promoting characteristics and biocontrol activities^14, 20, 21^. The molecular mechanism of FZB42 confers resistance to salt stress in plants is still unknown.

In the present study, we evaluated the effects of FZB42 inoculation on the growth of *A. thaliana* under salt stress (100 mM NaCl) and identified plant genes with important roles in response to this bacterium by transcriptome profiling in *A. thaliana* shoot tissues using Illumina sequencing. Our study is to characterize the plant transcriptome under salt stress after colonization by PGPR, thereby providing insights into the molecular mechanism that allows PGPR to induce salt tolerance in plants.

## Results

### *Bacillus amyloliquefaciens* FZB42 promoted Arabidopsis growth under non-salt stress and salt stress conditions

**Figure 1:**
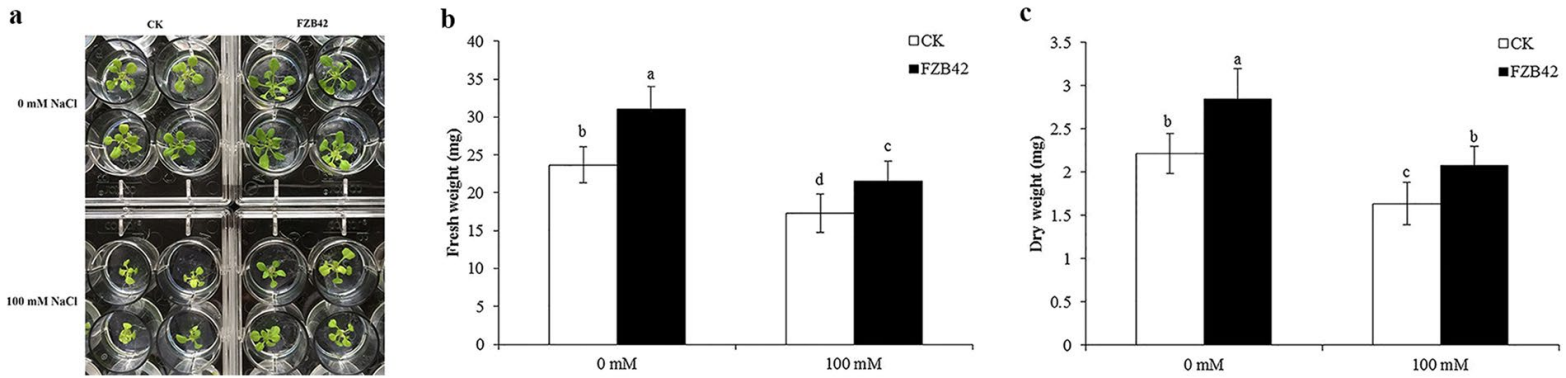
Effects of FZB42 on growth and salt tolerance in *Arabidopsis thaliana*. Plant seedlings were grown for 10 days in half-strength MS medium, before transplanting into 12-well microtiter plates. After treatment for 3 days with FZB42, NaCl was added to obtain a final concentration of 100 mM. At 10 days after bacterial treatment, the shoot parts were removed and measured to determine the fresh weight. The dry weight of plants was measured after in a drying oven at 75 °C for 2 days. (**a**) Photographs were taken of the plants after exposure to salt stress with or without FZB42. (**b**) FZB42 increased the fresh weight. (**c**) FZB42 increased the dry weight. White and black bars represent FZB42-inoculated and non-inoculated treatments, respectively. Error bars mean standard deviation. Different letters indicate statistically significant differences between treatments (Duncan’s multiple range tests, *P* < 0.05).

To confirm that FZB42 alleviated plant salt stress, plant growth was monitored in the presence or absence of 100 mM NaCl after inoculation with FZB42. After salt stress treatment for 10 days, the biomass of *Arabidopsis* seedlings inoculated with FZB42 was higher based on measurements of the fresh and dry weight of the shoot parts of plants (Fig. 1). Compared with non-inoculated seedlings, FZB42-inoculated *Arabidopsis* exhibited 31.2% and 24.7% increases in the plant fresh weight at 0 and 100 mM NaCl, respectively (Fig. 1b). Similarly, differences were also observed in the dry biomass accumulation, where FZB42-inoculated *Arabidopsis* exhibited 28.3% and 27.2% increases in the plant dry weight at 0 and 100 mM NaCl compared with non-inoculated seedlings, respectively (Fig. 1c). These results suggest that FZB42 promotes plant growth under non-stress and saline conditions.

### Characterization of the sequenced Illumina libraries

To identify genes related to FZB42-induced plant salt tolerance, twelve Col-0 Illumina libraries including three biological replicates for treatments were constructed from shoot tissues treated or untreated with FZB42 and grown at 0 and 100 mM NaCl. The FPKM (fragments per kilobase of exon per million fragments mapped) expression data was tested by correlation analysis to evaluate sampling between three biological replicates. All the correlation coefficiencies between biological pairs were over 0.98 (Supplementary Fig. S1). An average raw reads about 27,673,695 with paired-ends of 150 bp were obtained from twelve samples, which comprised 24,612,344, 23,716,667, 33,409,535, and 24,784,139 clean reads (paired-ends), respectively (Supplementary Table S1). After mapping the clean reads obtained from the twelve libraries onto the *Arabidopsis* genome, at least 83% of the clean reads could be mapped onto the reference database. The proportion of uniquely mapped reads was over 76.8%, whereas multiple mapped reads were found mainly in the rRNA or intergenic regions. The mapped reads from the four samples were distributed mainly in exon regions, followed by intergenic and intron regions (Supplementary Fig. S2). Sequencing saturation was analyzed to assess whether the sequencing depth was sufficient for transcriptome coverage. The results showed that the number of detected genes was saturated when the total reads ≥2 million (Supplementary Fig. S3), thereby satisfying the requirements for further analysis.

### KEGG pathways

**Figure 2:**
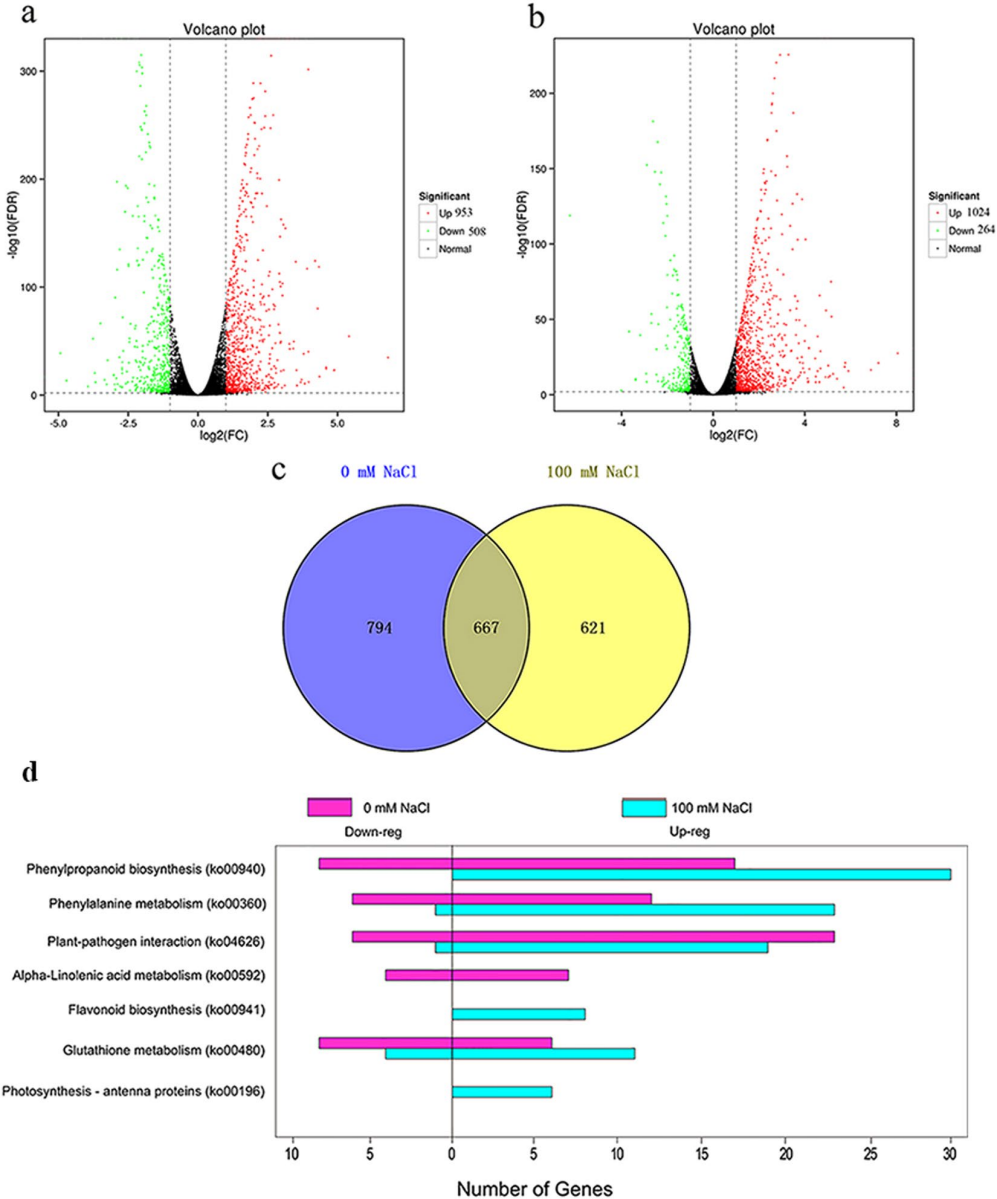
Differentially expressed genes in FZB42-inoculated versus non-inoculated plants at 0 and 100 mM NaCl. The volcano plot shows the differentially expressed genes (DEGs) with 0 mM NaCl (**a**) and 100 mM NaCl (**b**) for FZB42-inoculated versus non-inoculated plants. DEGs were identified using a threshold of FDR ≤ 0.01 and |log_2_ (fold change)| ≥ 1. The red dots represent upregulated DEGs and the green dots indicate downregulated DEGs. The black dots show genes without obvious changes between two corresponding samples. The Venn diagram shows the number of specific and common DEGs in FZB42-inoculated versus non-inoculated plants at 0 and 100 mM NaCl (**c**) (see Supplementary Table S2). Pathway enrichment analysis for DEGs. The gene numbers with significant enrichment are shown for upregulated and downregulated genes at 0 and 100 mM NaCl together (Corrected_*P*-value < 0.05) (**d**) (see Supplementary Table S3).

To elucidate the molecular mechanisms associated with the plant response to FZB42 under salt stress conditions, we identified the differentially expressed genes (DEGs) in *A. thaliana* under the four experimental treatments. Compared with non-inoculated plants, there were 1461 and 1288 DEGs at 0 and 100 mM NaCl, respectively, where 953 were upregulated and 508 were downregulated at 0 mM NaCl, and 1024 were upregulated and 264 were downregulated at 100 mM NaCl (Fig. 2a,b). In total, 794 genes were specifically regulated at 0 mM NaCl and 621 DEGs were identified only under salt stress condition (Fig. 2c) (Supplementary Table S2).

To further understand the biological functions of the DEGs, all of the annotated genes were mapped onto terms in the Kyoto Encyclopedia of Genes and Genomes (KEGG) database to search for significantly enriched genes involved in specific metabolic pathways. At 0 mM NaCl, 402 of 1461 DEGs mapped onto the KEGG database for *Arabidopsis* (Supplementary Fig. S4a) and 5 terms were significantly enriched (Corrected *P*-value < 0.05). Similarly, at 100 mM NaCl, 353 of 1288 DEGs mapped onto the KEGG database for *Arabidopsis* (Supplementary Fig. S4b) and six terms were significantly enriched (Corrected *P*-value < 0.05) (Fig. 2d, Supplementary Table S3). Four terms comprising phenylpropanoid biosynthesis, phenylalanine metabolism, glutathione metabolism, and plant-pathogen interaction were significantly enriched at both 0 and 100 mM NaCl. Interestingly, one term related to plant photosynthesis process, photosynthesis-antenna proteins, was only significantly enriched at 100 mM NaCl. Six genes in the photosynthesis-antenna proteins pathway were all upregulated, which suggested that FZB42 might enhance plant photosynthesis under salt stress compared with non-stress condition.

### Effects of FZB42 on the expression patterns of DEGs

**Figure 3:**
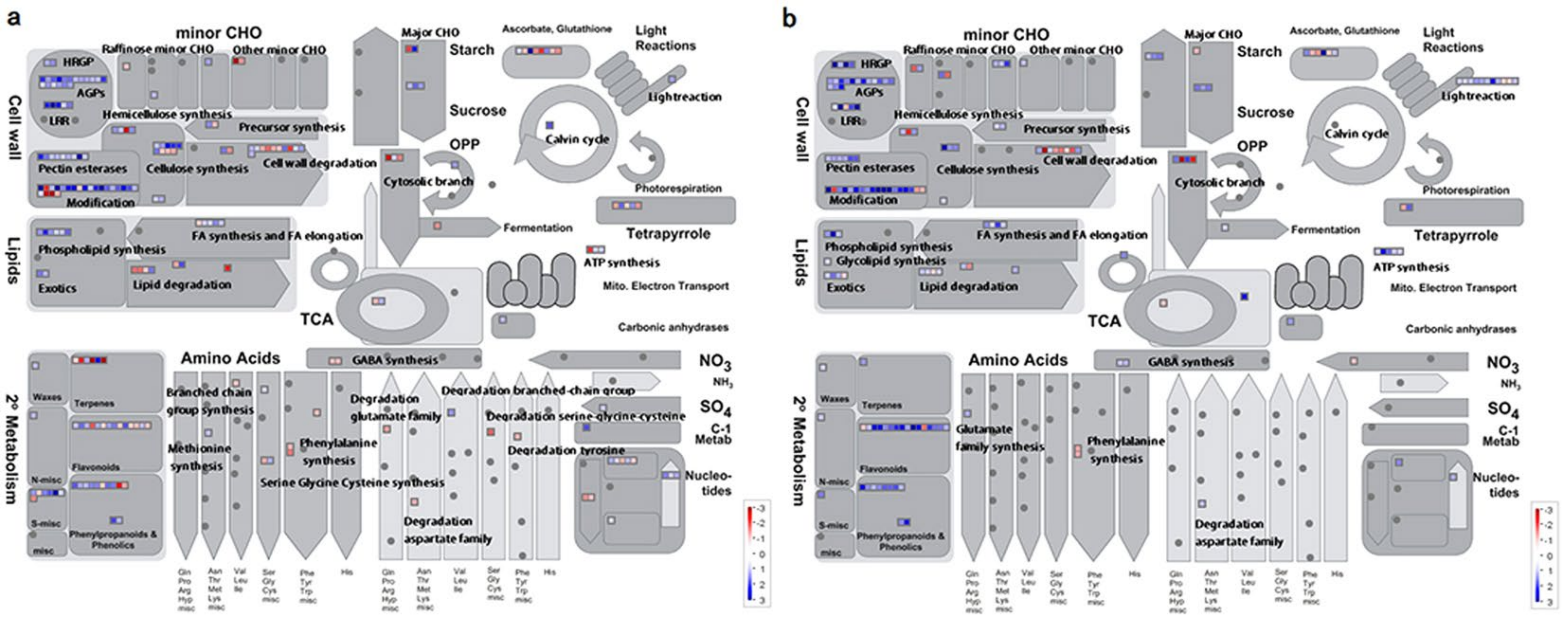
Schematic overview of differentially expressed genes (DEGs) related to different metabolic processes in the Mapman ontology. Upregulated and downregulated genes in FZB42-inoculated versus non-inoculated plants at 0 and 100 mM NaCl are shown in (**a,b**). Blue squares indicate upregulated DEGs and the red squares indicate downregulated DEGs. The scale bar is shown as log_2_.

**Figure 4:**
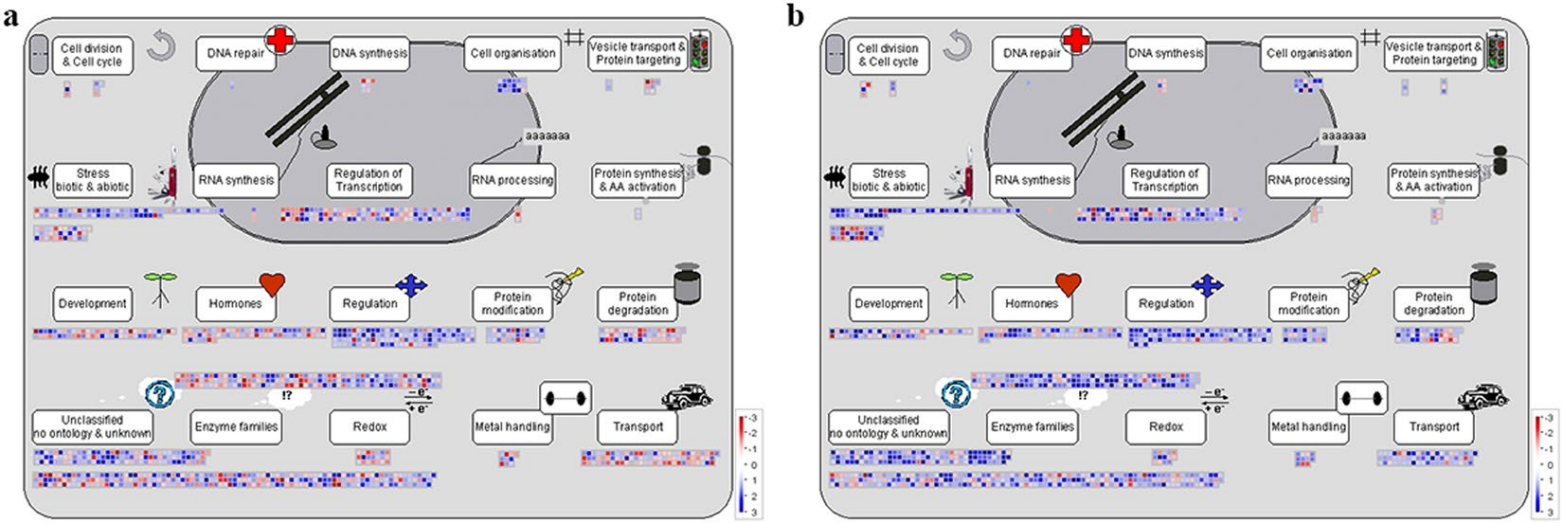
Overview of cell functions related to differentially expressed genes (DEGs) in FZB42-inoculated versus non-inoculated plants at 0 mM (**a**) and 100 mM NaCl (**b**) according to Mapman. Blue squares indicate upregulated DEGs and red squares indicate downregulated DEGs.

MapMan was used to integrate and visualize the DEGs according to their functions in metabolic pathways (Figs 3 and 4, Supplementary Tables S4 and S5). According to the *Arabidopsis* transcriptome database TAIR 10, two image annotator modules for overview and biological process were used to map BINs/subBINS data. This allowed us to explore the genes that are induced by FZB42-inoculation by focusing on genes related to energy metabolism, major and minor carbohydrate metabolism, hormone metabolism, redox, transcription factors (TFs), and stress responses^22^.

### Energy metabolism

An overview of the DEGs modulated by FZB42 according to their corresponding metabolic pathways was generated using MapMan (Fig. 3a,b). In total, Mapman mapped out of 1461 and 1288 DEGs, 203 and 180 were differentially mapped at 0 and 100 mM NaCl, respectively. FZB42 modified the expression pattern of some genes related to major and minor CHO metabolism. Compared with 0 mM NaCl, the numbers of upregulated genes related to the light reaction in photosynthesis and ATP synthesis were higher at 100 mM NaCl, suggesting that FZB42 might promote plants produce more energy under salt stress condition. These results were consistent with those shown in Fig. 2. Furthermore, several genes related to photosynthesis like the ones encoding for the photosynthetic pigments (tetrapyrroles) also showed differential expression patterns under the two conditions. However, one gene (ribulose-5-phosphate isomerase) encoding the enzyme of calvin cycle was only significantly induced to 3.8-fold at 0 mM NaCl (Supplementary Tables S4 and S6).

Several pathways related to the biosynthesis and degradation of amino acids and trehalose metabolism were also modulated by FZB42. Among them, we observed that delta1-pyrroline-5-carboxylate synthase 1 (*P5CS1*), a key enzyme in proline production, was only upregulated in FZB42-inoculated plants in the presence of salt (Fig. 3b). As an important osmoprotectant, proline was usually accumulated in plant cells when plants were in response to abiotic stresses. The positive regulation of *P5CS1* was conducive to the accumulation of proline, which helped plants to survive unfavourable salt conditions. For trehalose metabolism, at 0 mM NaCl, one gene (trehalose-6-phosphatase synthase S8, *ATTPS8*) was upregulated, whereas two genes were detected under salt stress condition, of which haloacid dehalogenase-like hydrolase (*HAD*) superfamily protein (*TPPH*) showed positive and trehalose-6-phosphate phosphatase (*ATTPPB*) showed negative regulation (Supplementary Tables S4 and S6).

### Genes associated with growth and development

At 100 mM NaCl, 4/11 genes associated with genetic material replication and synthesis were upregulated, including ribonuclease, DNA synthesis, RNA synthesis, and purine permease (Fig. 4b), whereas 8/15 genes were upregulated at 0 mM NaCl (Fig. 4a). Furthermore, two DNA repair related genes (DNA repair protein and methyladenine glycosylase family protein) and one DNA repair related gene (methyladenine glycosylase family protein) were all upregulated at 0 and 100 mM NaCl, respectively (Supplementary Tables S5 and S6).

Similarly, the transcript levels of several genes involved in plant growth-related processes also increased in FZB42-inoculated seedlings at 0 and 100 mM NaCl, including extensins (i.e., proline-rich extensin-like family protein and expansin-like proteins), cell wall-modification enzymes (i.e., pectinesterase and polygalacturonase). Polygalacturonase is an enzyme that catalyzes carbohydrate synthesis, which is an important component of the pectin network that comprises plant cell walls. In addition, genes encoding other cell wall modification enzymes (xyloglucan endotransglucosylase/hydrolase) that participate in cell wall loosening were also modified by FZB42. At 100 mM NaCl, ten transcripts encoding xyloglucan endotransglucosylase/hydrolase were all upregulated, whereas only 11/14 genes were upregulated at 0 mM NaCl. The upregulation of these genes might benefit the plant cell wall extension (Supplementary Tables S5 and S6).

In addition, two genes involved in the cell cycle (crooked neck protein and *CYCD4;1*) and one gene involved in cell division (mitogen-activated protein kinase kinase kinase 16) were upregulated by FZB42 at 100 mM NaCl (Fig. 4b). Four genes involved in the cell cycle and cell division were upregulated at 0 mM NaCl (Fig. 4a, Supplementary Tables S5 and S6). Changes in the transcription levels of cell wall modification and cell division-related genes are beneficial for cell development and division, thereby allowing plants grow stronger.

### Stress-response genes

We analyzed DEGs modified by abiotic and biotic stresses in plants inoculated with FZB42 at 0 and 100 mM NaCl (Fig. 4a,b, Supplementary Tables S5 and S6). Interestingly, compared with salt stress, more genes involved in biotic and abiotic stresses were differentially expressed by FZB42 at 0 mM NaCl.

At 0 mM NaCl, 34 abiotic stress-related genes and 67 biotic stress-related genes were detected, of which 21 and 59 genes were upregulated, respectively. Similarly, at 100 mM NaCl, 32 abiotic stress-related genes and 52 biotic stress-related genes were identified, of which 19 and 51 genes were significantly upregulated, respectively. Interestingly, 20 genes encoding PR-proteins were all upregulated at 100 mM NaCl whereas 36 PR-proteins genes were induced by FZB42 0 mM NaCl, of which 32 genes were upregulated. In addition, five genes responding to cold stress were only detected at 100 mM NaCl and all of them were upregulated by FZB42. While three genes responding to drought and salt were upregulated at 0 mM NaCl, two genes responding to drought and salt were identified at 100 mM NaCl and *AT5G04060* showed negative regulation and *AT4G30650* showed positive regulation by FZB42.

Heat-shock proteins (Hsps) are molecular chaperones and a ubiquitous feature of cells, which prevent the stress-induced denaturation of other proteins^23^. In this study, 18 and seventeen Hsps were differentially expressed in FZB42-inoculated plants at 0 and 100 mM NaCl. Interestingly, a lower number of genes (35.3%) was upregulated at 100 mM NaCl compared with 0 mM NaCl (50%).

Many transcripts encoding for antioxidases that function as reactive oxygen species (ROS) scavenger were also modified by FZB42. Our results showed genes involved in antioxidant responses such as glutathione-S-transferase (11 genes 54.5% of which were upregulated), peroxidases (10 genes 90% were upregulated), and redox (21 genes 38.1% of which were upregulated) were differentially regulated by FZB42 at 0 mM NaCl (Fig. 4a). An increase in the number of upregulated antioxidant-related genes was found at 100 mM NaCl compared with 0 mM NaCl. At 100 mM NaCl, genes involved in antioxidant responses such as glutathione-S-transferase (13 genes 84.6% of which were upregulated), peroxidases (18 genes 100% of which were upregulated, fold change ranged between 2.1 and 118), and redox (14 genes 57.1% of which were upregulated) were differentially regulated by FZB42 (Fig. 4b). In particular, the upregulated of the redox.thioredoxin was particularly found at 100 mM NaCl (Fig. 4b, Supplementary Tables S5 and S6).

### Hormone-related genes

The expression patterns of genes involved in jasmonic acid (JA), ethylene (ET), abscisic acid (ABA), auxin, salicylic acid (SA), and brassinosteroid, were differentially regulated by FZB42 at 0 and 100 mM NaCl (Fig. 4, Supplementary Tables S5 and S6).

### Jasmonic acid and Ethylene metabolism

Both JA and ET are considered to be stress-responsive phytohormones and they are capable of eliciting defence responses. Compared with 100 mM NaCl, the treatment with 0 mM NaCl produced a much higher number of DEGs related to JA and ET metabolism after FZB42-inoculation.

In terms of JA metabolism, three genes involved in JA synthesis were found to be upregulated by FZB42, including two lipoxygenases (*AT1G17420* and *AT1G72520*) and one allene oxide cyclase (*AT3G25780*), downstream of the lipoxygenases in JA synthesis, which had a fold change of 2.3, 2.8 and 2.6, respectively, compared with non-FZB42 inoculation under salt stress condition. In addition, three JA-responsive defense-related genes (*AT2G26010*, *AT5G44420*, and *At1g75830*), were also upregulated under salt stress condition. Four genes involved in JA synthesis were also found to be upregulated by FZB42 under non-stress condition, whereas jasmonic acid carboxyl methyltransferase (*JMT*), a key enzyme for jasmonate-regulated plant responses, and jacalin lectin family protein (*AT1G52100*) were downregulated (Fig. 4a,b, Supplementary Table S6).

Similar to JA, RNAseq data also suggestes that FZB42 induces ethylene biosynthesis under salt stress condition. Four ACC synthases (*ACS7*, *ACS2*, *ACS8*, and *ACS11*), the key enzymes in ethylene synthesis, were upregulated 4.4, 5, 5.1 and 3.3-fold by FZB42 at 100 mM NaCl. Likewise, 1-aminocyclopropane-1-carboxylate oxidase (*AT1G77330*) that plays an important role in converting the ACC into ethylene was upregulated 3.3-fold at 100 mM NaCl. Under salt stress condition, FZB42 also affected 5 ethylene signal transduction genes, all of which were upregulated except *HLS1* (*AT4G37580*). At 0 mM NaCl, two genes involved in ethylene biosynthesis were detected by FZB42, only one of which was upregulated. The transcriptional levels of seven ethylene signal transduction genes (71.4% upregulated) were also affected by FZB42 under non-stress condition (Fig. 4a,b, Supplementary Tables S5 and S6).

### Abscisic acid metabolism

9-cis epoxycarotenoid dioxygenase (*NCED*) genes encode key enzymes for abscisic acid biosynthesis^24^. In our study, one *NCED* gene (*NCED3*, *AT3G14440*) showed decreased expression level at both 0 and 100 mM NaCl. *NCED4* (*AT4G19170*) was only significantly downregulated at 100 mM NaCl. Specifically, two genes involved in abscisic acid signal transduction were only detected at 100 mM NaCl, both of which *ABI1* (*AT4G26080*) and *MARD1* (*AT3G63210*) showed negative regulation (Fig. 4a,b, Supplementary Tables S5 and S6), suggesting FZB42 conferred plant salt tolerance in ABA-independent pathway.

### Auxin metabolism

Two genes involved in auxin biosynthesis were identified in FZB42-inoculated plant at 0 mM NaCl, of which one (*TGG2*) was upregulated and one (*UGT1*) was downregulated. Similar to this, two genes involved in auxin biosynthesis were also detected in FZB42-inoculated plants at 100 mM NaCl, including *ILL5* (upregulated 54.4-fold) and *UGT1* (downregulated 2.3-fold). 16 and nineteen auxin induced-regulated-responsive-activated genes were identified in FZB42-inoculated plant at 0 mM and 100 mM NaCl, respectively, of which 10 and fifteen were differentially upregulated (Fig. 4a,b, Supplementary Tables S5 and S6).

### Salicylic acid metabolism

FZB42 affected the expression of 6 transcripts involved in SA synthesis, all of which were upregulated except *UGT74E2* (*AT1G05680*) and *GAMT2* (*AT5G56300*) at 0 mM NaCl. Intriguingly, 4 transcripts involved in SA synthesis were all upregulated at 100 mM NaCl (Fig. 4a,b, Supplementary Tables S5 and S6).

### Bassinosteroid metabolism

As plant steroidal, brassinosteroids (BRs) regulate plant growth and development. Overexpression of *CHI2/SHK1/SOB7* (*CYP72C1*, *At1g17060*) could cause BR deficiency^25, 26^. The transcriptional level of *CHI2/SHK1/SOB7* was downregulated by FZB42 at 0 mM and 100 mM NaCl, respectively. This indicated that BR might accumulate in FZB42-inoculated plants compared with non-inoculated plants. Furthermore, one gene *EXL5* (*AT2G17230*) involved bassinosteroid signal transduction was identified, which was differentially upregulated at both 0 and 100 mM NaCl. (Fig. 4a,b, Supplementary Tables S5 and S6).

### Transcription factors

In total, 58 (32 upregulated) and 49 (37 upregulated) differentially expressed TFs were identified in FZB42-inoculated plants at 0 mM and 100 mM NaCl, respectively (Fig. 4a,b, Supplementary Tables S5 and S6). Several TF families with pivotal roles in response to abiotic stress were differentially regulated, including MYB, WRKY, bZIP, AP2/EREBP, and bHLH. These included seven common DEGs from the MYB family comprising *MYB6*, *MYB77*, *MYB10*, *MYB3*, *MYBL2*, *MYBL*, and *RVE2*, as well as specific 16 at 0 mM NaCl and 8 at 100 mM NaCl. *MYB3*, *MYB6*, *MYB15*, *MYB74*, *MYB77*, and two MYB-related gene family CCA1-like genes (*CCA1* and *LHY*) except *MYB3* and one MYB-related gene family CCA1-like gene (*RVE2*) were all upregulated by FZB42 under salt stress condition; interestingly, all of these transcripts responded to ethylene and JA^27^. Furthermore, within the WRKY transcription factor family, 4 (100% upregulated) and twelve (91.7% upregulated) WRKY genes were obsered at 0 and 100 mM NaCl, respectively. Similarly, three WRKY transcriptional factors (TFs) (*WRKY 30*, 46 and 48) identified in this study under salt stress were also known to respond to ethylene^28, 29^, further pointing to a role of ethylene and JA in inducing salt tolerance conferred by FZB42. FZB42 affected the expression of 15 and 15 (*AP2/EREBPs*) at 0 mM and 100 mM NaCl, 26.7% and 60% of which were upregulated, respectively. All the five bZIPs except *HYH* and one bZIP (*AT5G04840*) were all upregulated at 0 and 100 mM NaCl, respectively. In addition, we also identified 11 (81.8% upregulated) and six (83.3% upregulated) bHLHs at 0 and 100 mM NaCl, respectively.

### FZB42 affected the Na^+^, K^+^, and Ca^2+^ balance under saline conditions

**Figure 5:**
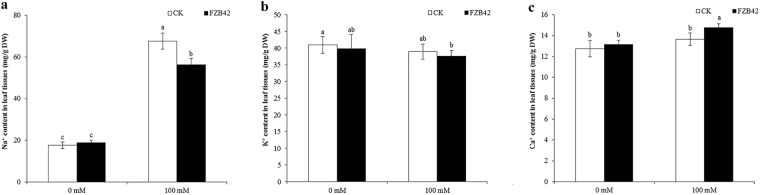
Effects of FZB42 on the ion contents of shoot tissues under salt stress. (**a**) Na^+^ content. (**b**) K^+^ content. (**c**) Ca^2+^ content. White and black bars represent FZB42-inoculated and non-inoculated treatments, respectively. Error bars mean standard deviation. Different letters indicate statistically significant differences between treatments (Duncan’s multiple range tests, *P* < 0.05).

We measured the ion contents of Na^+^, K^+^, and Ca^2+^, which are involved in photosynthesis and energy transfer. We found that all three of these ions exhibited greater changes in FZB42-inoculated plants compared with non-inoculated plants at 0 mM and 100 mM NaCl. And similar results were obtained by Ghaffari *et al*.^22^ studying root-colonized fungus *Piriformospora indica* induced barley salt stress tolerance. At 100 mM NaCl, the Na^+^ content of shoot tissues from FZB42-inoculated plants reduced by 16.4% compared with that from non-inoculated plants, which suggested that FZB42 decreased Na^+^ accumulation under salt stress condition (Fig. 5a). This result was consistent with the RNA-seq data, which detected three transcripts encoding Na^+^/H^+^ antiporter (*NHX1, CHX16*, and *CHX17*) and one transcript encoding sodium transporter (*HKT1*), and all of these four genes showed positive regulation. However, FZB42 had no effects on K^+^ accumulation, although there was only a slight decrease in the K^+^ content in FZB42-inoculated plants at both 0 and 100 mM NaCl (Fig. 5b). The K^+^/Na^+^ ratio in the shoot tissues was increased by FZB42 inoculation under salt stress condition, which was beneficial for salt adaptation by plants. The Ca^2+^ level was only significantly increased in FZB42-inoculated plants under salt stress condition (*P* < 0.05), despite the fact that a slightly high Ca^2+^ level was also detected in FZB42-inoculated plants under non-stress condition, but no significant difference was detected (Fig. 5c). Similar to the K^+^/Na^+^ ratio, there was a higher Ca^2+^/Na^+^ ratio in FZB42-inoculated plants compared with non-inoculated plants under salt stress condition.

### Inducible proline accumulation

The accumulation of osmoprotectants, including glycine betaine, trehalose, and proline, is a common stress avoidance mechanism in plants such as *Arabidopsis*. To investigate whether proline accumulation was facilitated by FZB42 under salt stress, the proline contents of *Arabidopsis* shoots were measured in the four treatments. In agreement with its transcriptional regulation, the highly upregulated expression of the key proline synthesis enzyme *P5CS1* showed that FZB42 could significantly enhance the accumulation of proline in plants treated with 100 mM NaCl. Under salt stress condition, the proline accumulation increased by over 32% when inoculated with FZB42 (79.3 ± 2.9 μg/g shoot) compared with non-inoculated seedlings (60.1 ± 2.7 μg/g shoot). The induced accumulation of proline by FZB42 helped Arabidopsis to resist the osmotic stress produced by high salinity. In addition, there was no significant difference in the amount of proline accumulation between FZB42-inoculated and non-inoculated plants under non-stress condition.

### Mutant analysis confirms the role of ET, JA and ABA in salt tolerance

**Figure 6:**
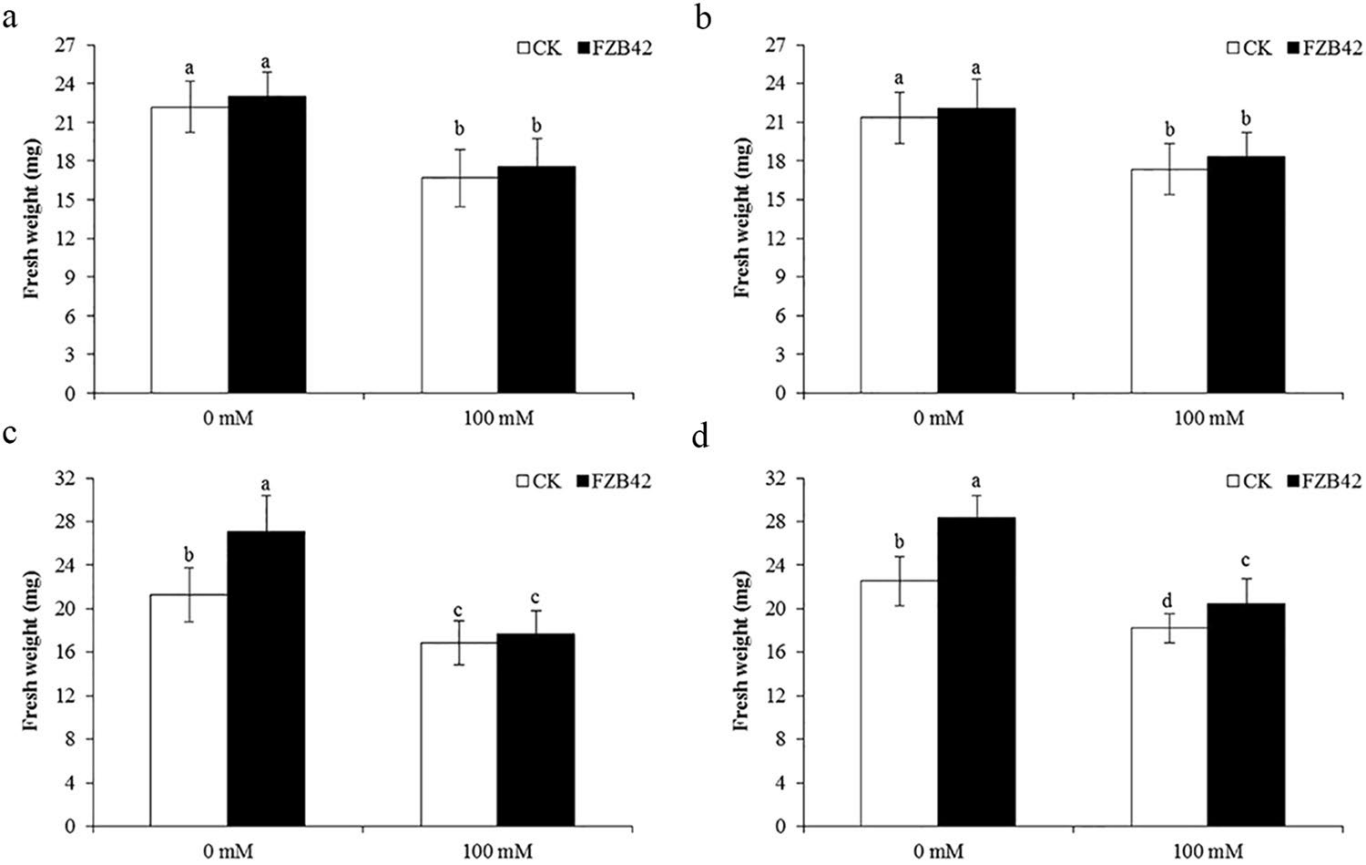
Effects of FZB42 on growth and salt tolerance in different *Arabidopsis* mutants. Four mutants were grown for 10 days in half-strength MS medium, before transplanting into 12-well microtiter plates. After treatment for 3 days with FZB42, NaCl was added to obtain a final concentration of 100 mM. At 10 days after bacterial treatment, the shoot parts were removed and measured to determine fresh weights. (**a**) *etr1-3* mutants. (**b**) *eto1* mutants. (**c**) *jar1-1* mutants. (**d**) *abi4-102* mutants. White and black bars represent FZB42-inoculated and non-inoculated treatments, respectively. Error bars mean standard deviation. Different letters indicate statistically significant differences between treatments (Duncan’s multiple range tests, *P* < 0.05).

In this study, we found 15 ethylene-responsive transcriptional factors were differentially regulated in response to FZB42 inoculation under salt stress. To further confirm the role of ethylene in FZB42 inducing plants salt tolerance, thus, Arabidopsis mutants *etr1-3* (defective in ethylene signaling) and *eto1* (ethylene overproducing mutant) were further analyzed for salt tolerance. It appeared that FZB42 did not promote *etr1-3* mutants growth at both 0 and 100 mM NaCl. Although the shoot weights of *etr1-3* mutants were slightly increased in FZB42-inoculated mutants than those of non-inoculated mutants, no significant differences were detected (Fig. 6a). Interestingly, similar results were also obtained in the ethylene-overproducing mutants eto1, FZB42 did not promote growth in *eto1* mutants under non-stress and stress conditions (Fig. 6b). The results of *etr1-3* and eto1 mutants indicated that ethylene signaling might be required for FZB42-plant growth promotion and inducing salt tolerance but a fine regulation of ethylene homeostasis was also necessary in this process. Likewise, JA insensitive mutants *jar1-1* treated with FZB42 also showed no significant tolerance to salt than non-inoculated mutants under stress condition, whereas significant shoot weight was detected in FZB42-inoculated mutants compared with non-inoculated plants at 0 mM NaCl (Fig. 6c). Lastly, *abi4-102* mutants (abscisic acid insensitive) inoculated by FZB42 exhibited better growth, as judged from shoot weight. There were significant differences in shoot weight between FZB42-inoculated and non-inoculated *abi4-102* mutants under both non-stress and stress conditions (Fig. 6d). These results were in agreement with our transcriptome data, genes related to ethylene and jasmonic acid synthesis were all upregulated by FZB42, whereas genes related to abscisic acid synthesis were all downregulated by FZB42 at 100 m M NaCl (Fig. 4b, Supplementary Tables 5 and S6). Taken together, the mutant analysis confirms that FZB42 might induce systemic salt tolerance via activating plant ET/JA signaling but not ABA-dependent pathway.

### Validation of DGEs by qRT-PCR

**Figure 7:**
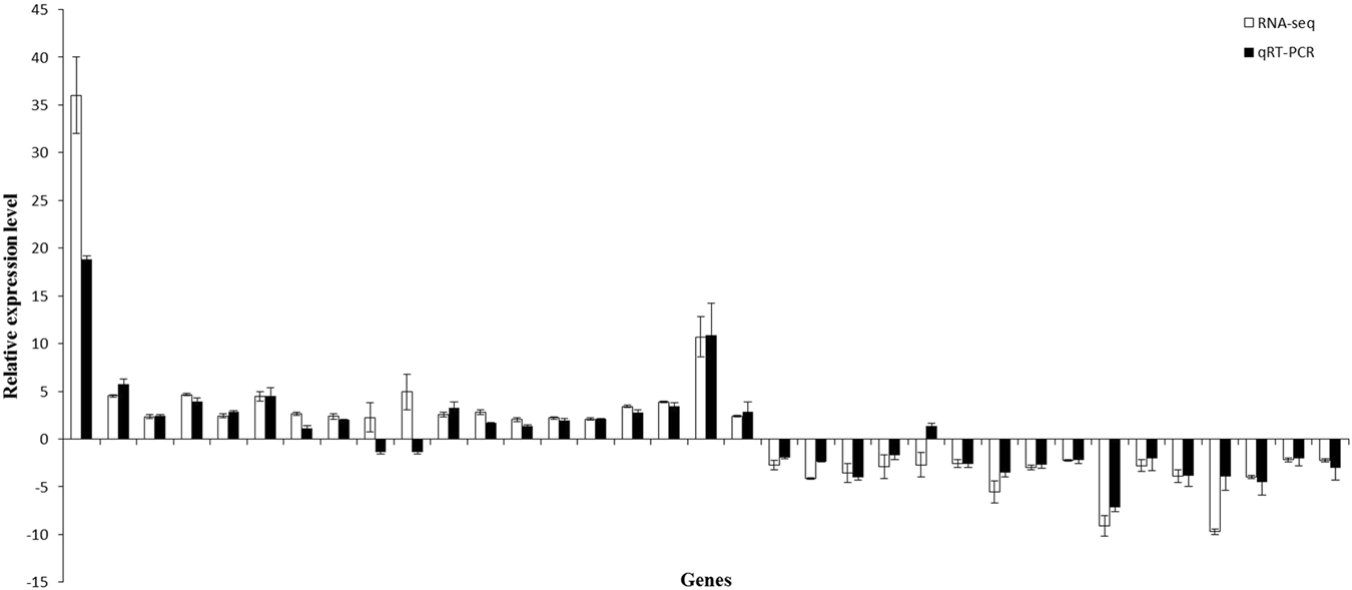
Comparisons of the relative expression levels determined by qRT-PCR (black bars) and RNA-seq (white bars) for 35 selected DEGs. The y-axis shows the relative expression levels of 35 selected genes. The x-axis indicates the 35 selected genes (from left to right: AT5G44420, AT3G23250, AT4g26200, AT1g17420, AT4G39210, AT4G23700, AT1g29910, AT2G05070, ATCG00120, ATCG00580, AT2G39800, AT2G05100, AT2G34420, AT3G27690, AT5G54270, AT4G39770, AT1G64170, AT1G14540, AT1G70290, AT3g14440, AT4G13250, AT1G78090, AT4G36110, AT5G23370, AT3G24500, AT3G03480, AT4G08390, AT1G05560, AT5G51720, AT4G32810, AT3G12580, AT3G04800, AT1G64900, AT3G44990, and AT1G10070). Bars represent the mean ± SD based on three replicates.

To validate the RNA-seq results, 35 selected DEGs were analyzed by qRT-PCR using specific primers (Supplementary Table S7). Among the 35 selected DEGs, nineteen were upregulated (fold change ranged between 2 and 36) and sixteen were downregulated (fold change ranged between −9.7 and −2.2). Most of the qRT-PCR results obtained from the 35 selected genes were in general agreement with their corresponding changes in transcript abundance according to RNA-seq (Fig. 7), thereby confirming the reliability of the Illumina results.

## Discussion

Previous studies have shown that PGPRs can elicit induced systemic resistance (ISR) to reduce the susceptibility of plants to diseases caused by biotic stresses, such as pathogenic fungi, bacteria, viruses, and nematodes^30^. In addition, some PGPRs also confer plants induced systemic tolerance to abiotic stresses such as salinity and drought^31^. The present study demonstrated that FZB42 alleviated salt stress in *Arabidopsis* and improved its growth under hydroponic conditions (Fig. 1), as shown in previous studies^3, 18, 32, 33^. Illumina sequencing was used to identify genes induced by FZB42 in *Arabidopsis* shoot tissues in response to salt stress.

Salinity usually causes ion toxicity due to ionic imbalance with a higher Na^+^ content in plant tissues. Thus, reducing the Na^+^ content is important for improving salt tolerance. Once Na^+^ influxes into plant cells, Na^+^/H^+^ antiporters are usually induced for Na^+^ recirculation and sequestration. It has been shown that *Arabidopsis NHX1* encodes a tonoplast Na^+^/H^+^ antiporter that is responsible for Na^+^ sequestration and it’s overexpression enhances Arabidopsis salt tolerance^34, 35^. In this study, *NHX1* was upregualted by FZB42 at 100 mM NaCl, indicating that FZB42 could alleviate alleviates salt stress in Arabidopsis by increasing vacuolar Na^+^ compartmentalization and then minimizing toxic ion accumulation (Fig. 5a). The expressions of *NHX1* in maize and Arabidopsis were also significantly induced by inoculation with *Bacillus amyloliquefaciens* SQR9, a close relative of FZB42, under salt stress condition^33, 36^. However, this result was contrary to the observed repression of NHX1 in rice inoculated with *Bacillus amyloliquefaciens* NBRISN13 and grown under salt stress^37^. That was probably due to the differences in plant species and sample collection time. The *HKT1* gene, a high-affinity K^+^ transporter, is known to be differentially induced in plant roots and shoots by volatiles emitted by *Bacillus subtilis* GB03, thereby reducing accumulation of Na^+^ throughout plant tissues^18^. Interestingly, *HKT1* was also significantly induced by FZB42 and SQR9 through root colonization under salt stress^33^, showing that *Bacillus amyloliquefaciens* could alleviate Na^+^ toxicity through regulating *HKT1* expression. Moreover, many studies showed that the exopolysaccharides (EPSs) produced by PGPRs could bind Na^+^ and subsequently decreased the amount of Na^+^ available for plant uptake^38–40^, and many EPS-producing PGPRs have been used to help plants alleviating salt stress^41, 42^. We also found that FZB42 adhered aggressively to Arabidopsis roots via biofilm formation (Supplementary Fig. S5). Thus, EPSs in the biofilm on Arabidopsis roots might also play important roles in binding Na^+^ to restrict the uptake of Na^+^ and ultimately decrease the plant Na^+^ content.

Salinity usually causes an increased level of ethylene in plants that ultimately inhibit plants growth^43^. Some PGPRs containing ACC deaminase that can sequester ACC (the immediate precursor of ethylene) have been documented to enhance plants salt tolerance via a reduction in levels of ethylene and consequently facilitate the growth of many crops such as rice, tomato, cotton, and maize under salinity condition^44–47^. And there are growing evidences showing that certain PGPRs without ACCD also induce plant salt tolerance; for example, although *Bacillus pumilus* WP8 and *Pseudomonas putida* RBP1 had no ACCD, they did successfully increase tomato salt tolerance^48^. SQR9 that failed to secrete ACCD also enhanced maize, Zea mays and Arabidopsis salt tolerance^33^. These findings implied that these PGPRs were not via reducing ethylene production to confer plant salt tolerance, supporting the view that reducing ethylene production is not the sole approach accounting for salt tolerace in all PGPRs. In contrast, low concentrations of ethylene appeared to enhance plant growth^49^. And several previous studies showed ethylene signaling was required for certain PGPRs-growth promotion. Chen *et al*.^50^ showed that *Variovorax paradoxus* 5C-2 promoted growth of ethylene overproducing mutant (*eto1-1*), but growth promotion was not found in ethylene-insensitive mutants (*etr1-1* and *ein2-1*). Poupin *et al*.^51^ found that *Burkholderia phytofirmans* PsJN-growth promotion was also related to ethylene signaling because it failed to stimulate the development of Arabidopsis mutants with an impaired ethylene (*ein2-1*). In this study, genes related to ethylene synthesis were differentially modified by FZB42 under non-stress and stress conditions. It was noted that five genes including (*ACS2*, *ACS7*, *ACS8*, *ACS11*, and *ACO5*) were all upregulated by FZB42 at 100 mM NaCl (Fig. 4b). On the other hand, mutants data showed FZB42 did not promote growth in *etr1-1* and *eto1* mutants (Fig. 6a,b) under non-stress and stress conditions. All of these results indicated that ethylene signaling might play important roles not only in FZB42-growth promotion but also in inducing plants salt tolerance but depending a fine ethylene homeostasis.

Jasmonic acid is potent signal compounds, and it accumulates rapidly when plants are under abiotic stress^52^. Many studies showed that JA is in response to salt stress, acting as positive regulators of salt tolerance^53, 54^; for example, salt tolerant tomato showed increased levels of jasmonic acid and JA content changed in response to salt-stress^55^. In this study, all of the genes involved in jasmonic acid synthesis were differentially upregulated by FZB42 at 0 and 100 mM NaCl. On the other hand, similar to ethylene mutants, FZB42 was also unable to induce salt tolerance in *jar1-1* mutant line of Arabidopsis (Fig. 6c) at 100 mM NaCl. The results of this study collectively suggested that FZB42 might use the ethylene and jasmonic acid transduction pathway to induce Arabidopsis salt systemic tolerance. This result was in agreement with the previous study done by Cho *et al*.^56^, who showed that that jasmonic acid and ethylene-regulated defense genes might play important roles in *Pseudomonas chlororaphis* O6 mediating systemic tolerance against abiotic and biotic stresses.

As important phytohormones, both JA and ET are considered to be stress-responsive hormones^57^, which can activate the plant defence network by regulating the expression of many TFs. Indeed, several TFs were identified in our transcriptomic analysis (Fig. 4a,b), including ERF, MYB, and WRKY. And most of these TFs played critical roles in biotic and abiotic stress responses in plants^58^. ERF TFs are located downstream of genes in the ET signaling pathway. The overexpression of many of these ERF genes conferred tolerance to various abiotic stresses in plants^59, 60^. Several MYB and WRKY TFs that respond to ET and JA were also upregulated by FZB42 under salt stress, further suggesting that JA/ET signaling might be involved in plant mechanism for salt stress adaptation conferred by FZB42.

The accumulation of osmoprotectants, including glycine betaine, trehalose, and proline, is a common stress avoidance mechanism in plants such as *Arabidopsis*. TSS and proline contents were highly enhanced in wheat treated with *Bacillus subtilis* SU47 and *Arthrobacter* sp. SU18 under salt stress^17^. The expression of proline biosynthetic gene *P5CS1* was highly upregulated in *Enterobacter* sp. EJ01 infected seedlings under salt condition^3^. Similar to the results of Kim *et al*.^3^, *P5CS1* was significantly upregulated by FZB42 under salt stress condition (Fig. 4b). Moreover, a highly proline content was also found in FZB42 treated plants. The increased amount of proline may function as an osmolyte or reactive oxygen species (ROS) scavenger, and its induced production by FZB42 under salt stress agreed with similar previous findings in cotton, rice, tomato, and *Arabidopsis* ^3, 30, 61^.

Abiotic stresses such as salt and drought can induce oxidative stress by generating ROS, which are scavenged by plants antioxidases such as catalase, superoxide dismutase, peroxidase, glutathione-S-transferase, and redox enzymes^62^. Researches with application of PGPR showed that PGPRs could significantly increase antioxidase activities of plants. Tomato treated with *Enterobacter* sp. EJ01 exhibited higher APX (ascorbate peroxidase) activities under salt condition than did EJ01-free seedlings^3^. The activities of POD and CAT was highly enhanced in *B. amyloliquefaciens* SQR9 treated maize than that of SQR9-free maize under salt stress^33^. In the current study, the enhanced expression levels of genes related to peroxidases, glutathione-S-transferases, and redox in FZB42-inoculated plants compared with non-inoculated plants showed that FZB42 could enhance the capacity of plants for scavenging ROS.

**Figure 8:**
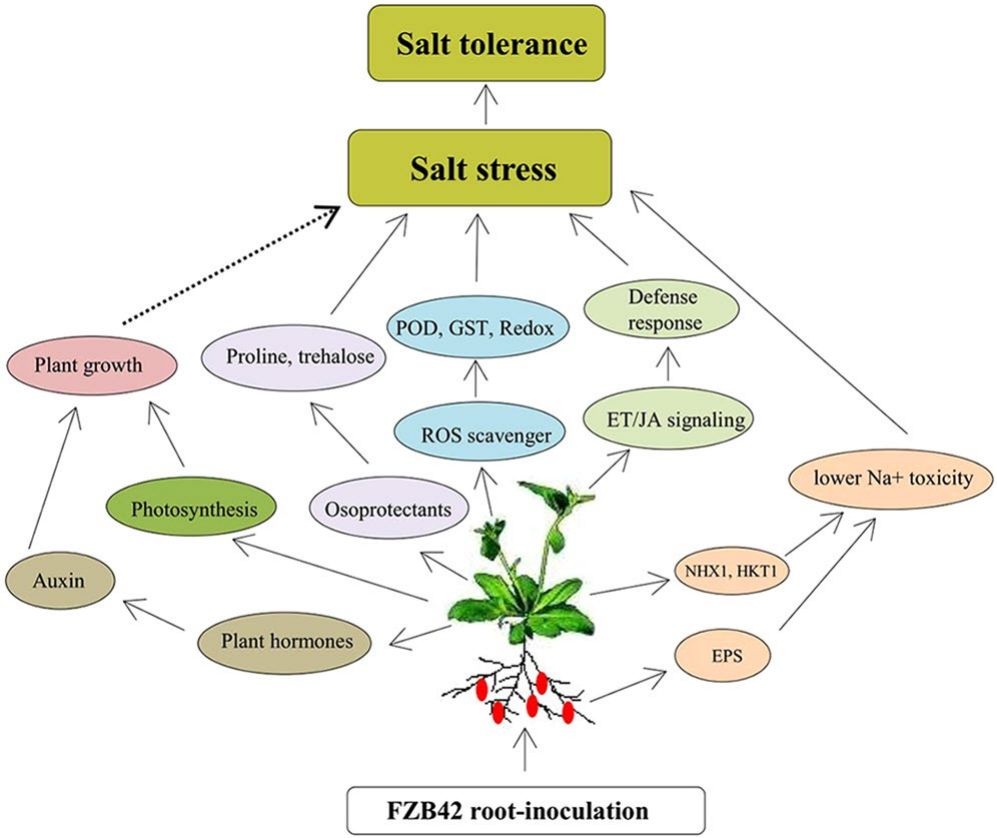
Pathways of FZB42 induced plants salt tolerance. Solid arrows indicated plant compounds or genes affected by FZB42, which had direct effects in enhancing plants salt tolerance. Broken arrows indicated plant growth affected by FZB42, which had indirect effects in enhancing plants salt tolerance. FZB42 root-inoculation enhanced plants photosynthesis and altered the expression levels of genes related to auxin, which promoted plants growing stronger and then enhanced plants tolerance to salt.

In conclusion, this study demonstrated that FZB42 can improve plant growth and enhance salt tolerance. In order to identify genes with important roles in the response to FZB42, we investigated the transcriptome profiles of *Arabidopsis* shoot tissues under salt stress using Illumina sequencing while the root samples were also collected for further analysis. The RNA-seq data indicated that FZB42 induced the upregulation of genes related to photosynthesis, auxin, ROS scavenging, Na^+^ translocation, osmoprotectants such as trehalose and proline as well as ethylene and jasmonic acid signaling under salt stress condition, thereby suggesting that these pathways might have roles in plant salt tolerance induced by FZB42 (Fig. 8). The results of this study provide a useful resource that may facilitate plant functional genomic studies, including many candidate genes as potential markers of tolerance to salt stress.

## Methods

### Plant materials and growth conditions

*Arabidopsis thaliana* (Col-0) seeds were surface sterilized with 2% NaClO for 3–4 min, washed five times with sterile water, and then planted on Petri dishes containing half-strength Murashige and Skoog (MS) solid medium, 0.8% (wt/vol) agar, and 1.5% (wt/vol) sucrose (pH 5.8) for germination. The growth conditions comprised 22 °C with a 16/8 h light/dark photoperiod.

### Culture conditions for *B. amyloliquefaciens* FZB42

*B. amyloliquefaciens* FZB42 (deposited as strain 10A6 in the culture collection at the Bacillus Genetic Stock Center) was grown in Luria-Bertani liquid medium at 37 °C with shaking at 200 rpm. After incubating overnight, cells were obtained by centrifugation at 10,000 × *g* for 6 min, washed once, and re-suspended in sterile water to 5.0 × 10^7^ colony-forming units/mL for use as an inoculum.

### Hydroponic culture and salt stress

Ten-day-old seedlings at appropriate growth states were transplanted singly into the wells of 12-well microtiter plates containing half-strength MS medium without sugar, and the medium was replaced every 3 days. Three days after transplantation, a half of 12-well microtiter plates was treated with FZB42 with a final concentration of 10^6^ CFUs ml^−1^ (B: only FZB42 inoculation); and the other half was treated with an equal amount of water as the non-inoculated control (CK: only water). At 3 days after bacterial inoculation, a half of 12-well microtiter plates (FZB42-inoculated and non-inoculated) was supplemented with 100 mM NaCl (B + S: 100 mM NaCl + FZB42 inoculation; S: only 100 mM NaCl). After salt treatment for 7 days, the shoot parts of seedlings were harvested to obtain physiological measurements and then frozen in liquid nitrogen, before storing at −80 °C until RNA extraction. This process was repeated three times as three biological replicates and each replicate included 72 plants.

### RNA isolation and Illumina sequencing

Total RNA was extracted from shoot tissues of 30 plants of three independent biological replicates using an RNeasy Plant Mini Kit according to the manufacturer’s instructions. The quality of RNA samples was assessed by UV-absorbance spectrophotometry and 1% agarose gel electrophoresis. After using OligodT magnetic beads to enrich poly(A) + mRNA, random hexamer primers were employed to synthesize cDNA. Finally, sequencing adaptors were ligated to the short fragments and suitable cDNA fragments were selected and purified. Subsequently, the final cDNA library templates from the four treatments were obtained by PCR. Sequencing was performed by Beijing Biomarker Technologies Corporation (Beijing, China) using a HiSeq. 2000 system (Illumina).

### Analysis of Illumina sequencing results

After removing adaptors, the unknown nucleotides and those of low quality were filtered from the raw reads to obtain clean reads, which were then mapped onto the *Arabidopsis thaliana* reference genome version TAIR10. Gene expression levels were calculated using FPKM values (fragments per kilobase of exon per million fragments mapped) by the Cufflinks software^63^. Genes with a false discovery rate (FDR) < 0.01 and |log_2_(fold change)| ≥ 1 were defined as differentially expressed genes (DEGs). DEGs were aligned to the Kyoto Encyclopedia of Genes and Genomes (KEGG) using BLASTX. Furthermore, the Mapman system was used to classify the regulated transcripts at 0 and 100 mM NaCl.

### Shoot tissues proline quantification

The shoot tissues proline content was quantified according to a previously described method^64^ with some slight modifications, as follows. First, 100 mg of shoot tissues were homogenized in 10 mL of 3% aqueous sulfosalicylic acid and filtered through filter paper. Next, 2 mL of the filtrate was reacted with 2 mL acid-ninhydrin and 2 mL glacial acetic acid in a 15 mL centrifuge tube for 1 h at 100 °C. The reaction mixture was then extracted with 4 mL of toluene for 15–20 s. The absorbance of the chromophore containing toluene aspirated from the aqueous phase was read at 520 nm using a spectrophotometer with toluene as the blank.

### Determination of Na^+^, K^+^, and Ca^2+^

Na^+^, K^+^, and Ca^2+^ were determined by inductively coupled plasma optical emission spectrometry (ICP-OES, PerkinElmer Optima 8000, USA). Shoot tissues were digested using HNO_3_ according to a previously described method^18^.

### Mutant analysis

Four kinds of Arabidopsis mutants *etr1-3* (ethylene insensitive mutant), *eto1* (ethylene overproducing mutant), *jar1-1* (jasmonate insensitive mutant) and *abi4-102* (abscisic acid insensitive mutant) donated by Clay were further studied for salt tolerance conferred by FZB42 by measuring the fresh weight of seedlings treated as described above.

### qRT-PCR analysis

qRT-PCR analysis was performed for 35 DGEs using *ACT2* (GenBank: *AT3G18780*) and *L2* (GenBank: *AT2G44065*) as genes to normalize the results. The primers used in this study are shown in Supplementary Table S7. First-strand cDNA synthesis was performed using a PrimeScript^TM^ RT reagent Kit with gDNA Eraser (perfect Real Time), which could remove genomic DNA. qRT-PCR was performed with a Mx3000 P system (Applied Biosystems) using SYBR^®^ Premix Ex Taq^™^ II (TliRNaseH Plus) (TaKaRa) according to the manufacturer’s instructions. qRT-PCR employed the following cycling conditions: 95 °C for 30 s, and 40 cycles at 95 °C for 5 s, 60 °C for 30 s, and 72 °C for 30 s, followed by extension. Each PCR analysis was repeated at least three times. The expression levels of genes were calculated using the threshold cycle2^−ΔΔCt^ method^65^.

### Statistical analysis

The data were analyzed by SAS 9.0 software. One-way ANOVA and Duncan multiple-range tests were performed to determine significant differences in four treatments.

## Electronic supplementary material

Supplementary data

Supplementary Table S2

Supplementary Table S4

Supplementary Table S5

Supplementary Table S6
