# Supplementary material for: Transcriptome profiling of genes involved in induced systemic salt tolerance conferred by Bacillus amyloliquefaciens FZB42 in Arabidopsis thaliana
Source: Sci Rep. 2017 Sep 13;7:10795. doi: 10.1038/s41598-017-11308-8 (PMC5597682; doi:10.1038/s41598-017-11308-8)
Supplement: Supplementary file 1 — Supplementary data [file 41598_2017_11308_MOESM1_ESM.pdf]

Title

Transcriptome profiling of genes involved in induced systemic salt tolerance conferred by *Bacillus amyloliquefaciens* FZB42 in *Arabidopsis thaliana*

Authors and e-mail address

Shaofang Liu[1,2], Haiting Hao[1,2], Xiang Lu[1,2], Xia Zhao[1,2], Yun Wang[3], Yubao Zhang[1,2], Zhongkui Xie[1,2], Ruoyu Wang[1,2*]

*Correspondence to: wangruoyu@lzb.ac.cn

Shaofang Liu[1,2] liushaofanghnpy@163.com

Haiting Hao[1,2] m18919046163@163.com

Xiang Lu[1,2] lux12@lzu.edu.en

Xia Zhao[1,2] zhaoxia@lzb.ac.cn

Yun Wang[3] hp.wangyun@gmail.com

Yubao Zhang[1,2] zyubao@yahoo.com

Zhongkui Xie[1,2] wxhcas@lzb.ac.cn

Ruoyu Wang[1,2*] wangruoyu@lzb.ac.cn

Affiliations

[1]Gaolan Station of Agricultural and Ecological Experiment, Northwest Institute of Eco-Environment and Resources, Chinese Academy of Sciences, Lanzhou, China.

[2]Key Laboratory of Stress Physiology and Ecology in Cold and Arid Regions of Gansu Province, Lanzhou, China.

[3]Key Laboratory of Desert and Desertification, Northwest Institute of Eco-Environment and Resources, Chinese Academy of Sciences, Lanzhou, Gansu 730000 China.

Full address for correspondence

*Corresponding author: Ruoyu Wang, Gaolan Station of Agricultural and Ecological Experiment, Northwest Institute of Eco-Environment and Resources, Chinese Academy of Sciences, Lanzhou, China.

Phone +86-931-4967205

E-mail address wangruoyu@lzb.ac.cn

Supplementary Information

This file contains 5 supplementary figures, three supplementary tables and captions of four supplementary tables that were uploaded as separate Excel documents.

Figure legends

Fig. S1. Distribution of correlation co-efficiencies between each pair of samples.

Fig. S2. Distribution of reads in the reference genome. (C-1, C-2, and C-3) only water. (S-1, S-2, and S-3) only 100 mM NaCl. (B-1, B-2, and B-3) only FZB42 inoculation. (B+S-1, B+S-2, and B+S-3) 100 mM NaCl + FZB42 inoculation.

Fig. S3. Sequencing saturation analysis. (C-1, C-2, and C-3) only water. (S-1, S-2, and S-3) only 100 mM NaCl. (B-1, B-2, and B-3) only FZB42 inoculation. (B+S-1, B+S-2, and B+S-3) 100 mM NaCl + FZB42 inoculation.

Fig. S4. Pathway enrichment analysis of DEGs. The gene number of enrichment of DEGs under 0 (a) and 100 mM NaCl (b) conditions.

Fig. S5 SEM micrograph of FZB42 colonizing *Arabidopsis* roots growing under salt stress.

Figure S1

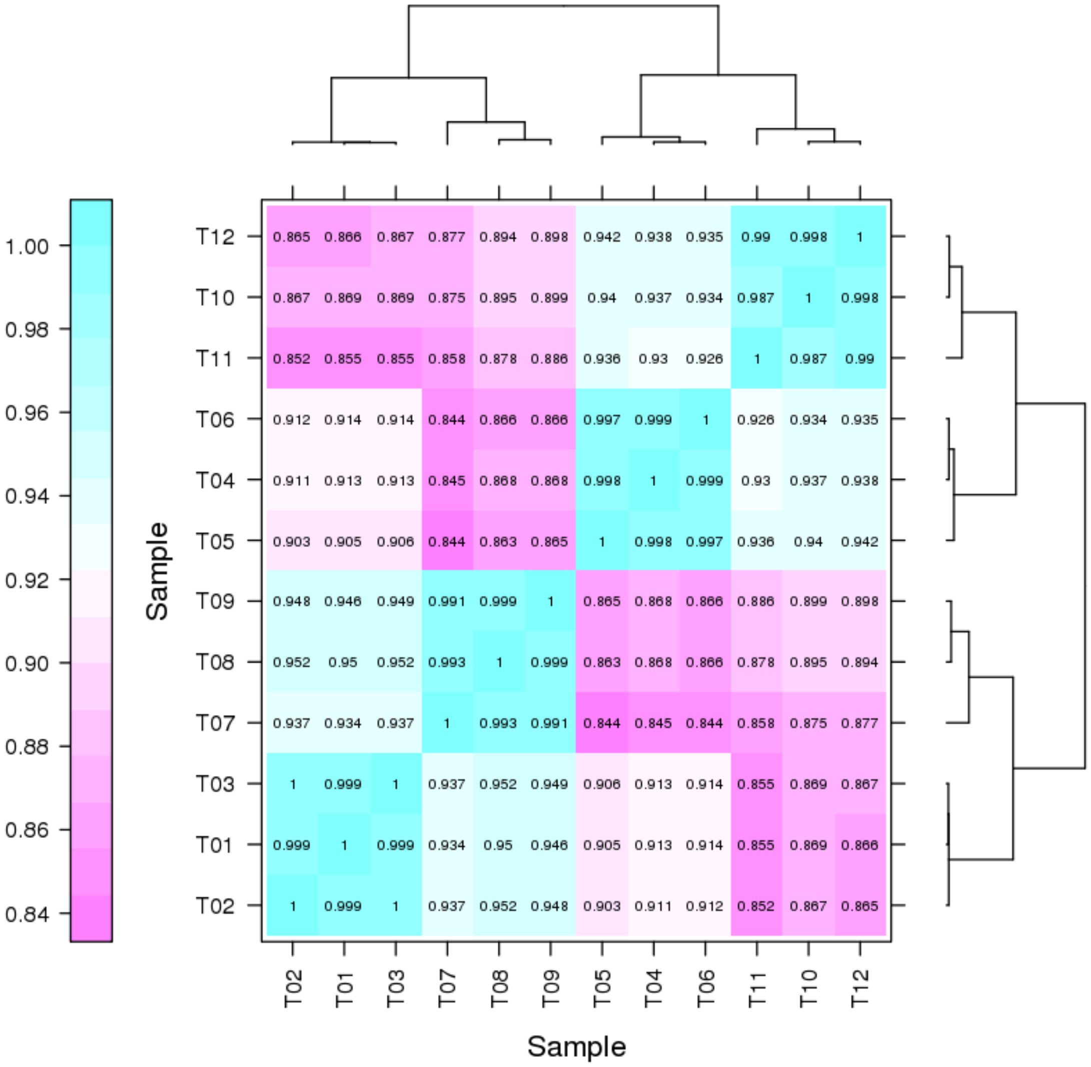
1.00
0.98
0.96
0.94
0.92
0.90
0.88
0.86
0.84
Sample
T12 0.865 0.866 0.867 0.877 0.894 0.898 0.942 0.938 0.935 0.99 0.998 1
T10 0.867 0.869 0.869 0.875 0.895 0.899 0.94 0.937 0.934 0.987 1 0.998
T11 0.852 0.855 0.855 0.858 0.878 0.886 0.936 0.93 0.926 1 0.987 0.99
T06 0.912 0.914 0.914 0.844 0.866 0.866 0.997 0.999 1 0.926 0.934 0.935
T04 0.911 0.913 0.913 0.845 0.868 0.868 0.998 1 0.999 0.93 0.937 0.938
T05 0.903 0.905 0.906 0.844 0.863 0.865 1 0.998 0.997 0.936 0.94 0.942
T09 0.948 0.946 0.949 0.991 0.999 1 0.865 0.868 0.866 0.886 0.899 0.898
T08 0.952 0.95 0.952 0.993 1 0.999 0.863 0.868 0.866 0.878 0.895 0.894
T07 0.937 0.934 0.937 1 0.993 0.991 0.844 0.845 0.844 0.858 0.875 0.877
T03 1 0.999 1 0.937 0.952 0.949 0.906 0.913 0.914 0.855 0.869 0.867
T01 0.999 1 0.999 0.934 0.95 0.946 0.905 0.913 0.914 0.855 0.869 0.866
T02 1 0.999 1 0.937 0.952 0.948 0.903 0.911 0.912 0.852 0.867 0.865
T02 T01 T03 T07 T08 T09 T05 T04 T06 T11 T10 T12
Sample

Figure S2

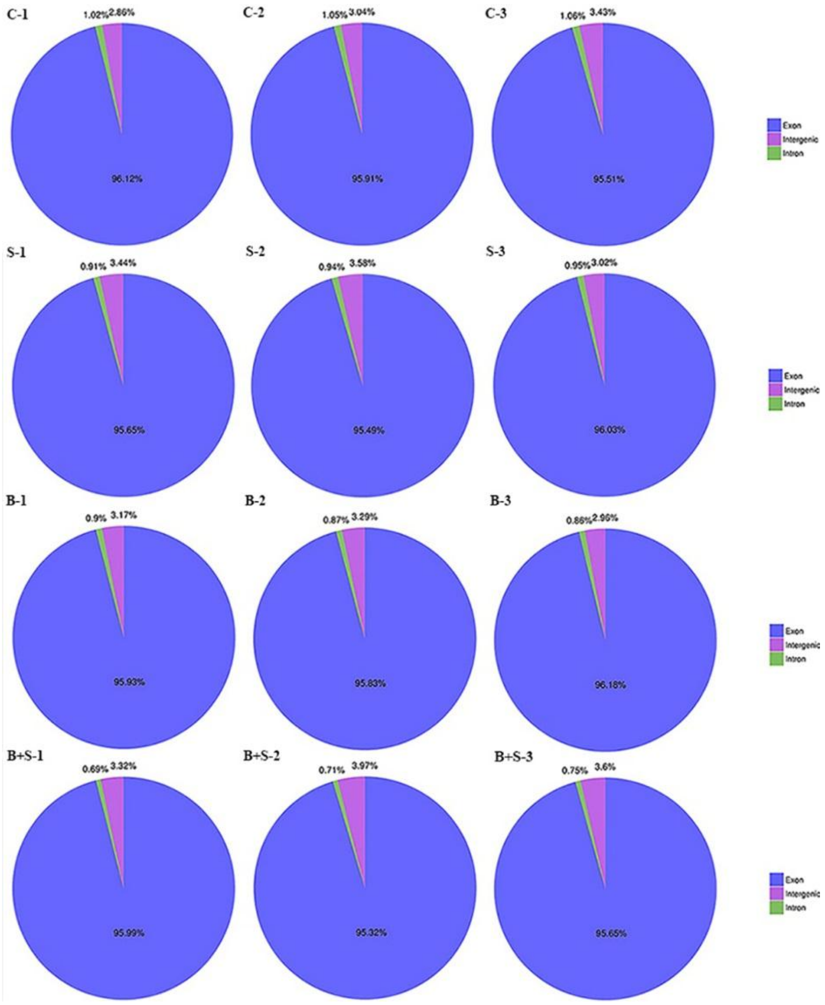

C-1
1.02%
2.86%
96.12%
C-2
1.05%
3.04%
95.91%
C-3
1.06%
3.43%
95.51%
S-1
0.91%
3.44%
95.65%
S-2
0.94%
3.58%
95.49%
S-3
0.95%
3.02%
96.03%
B-1
0.9%
3.17%
95.93%
B-2
0.87%
3.29%
95.83%
B-3
0.86%
2.96%
96.18%
B+S-1
0.69%
3.32%
95.99%
B+S-2
0.71%
3.97%
95.32%
B+S-3
0.75%
3.6%
95.65%
Exon
Intergenic
Intron

Figure S3

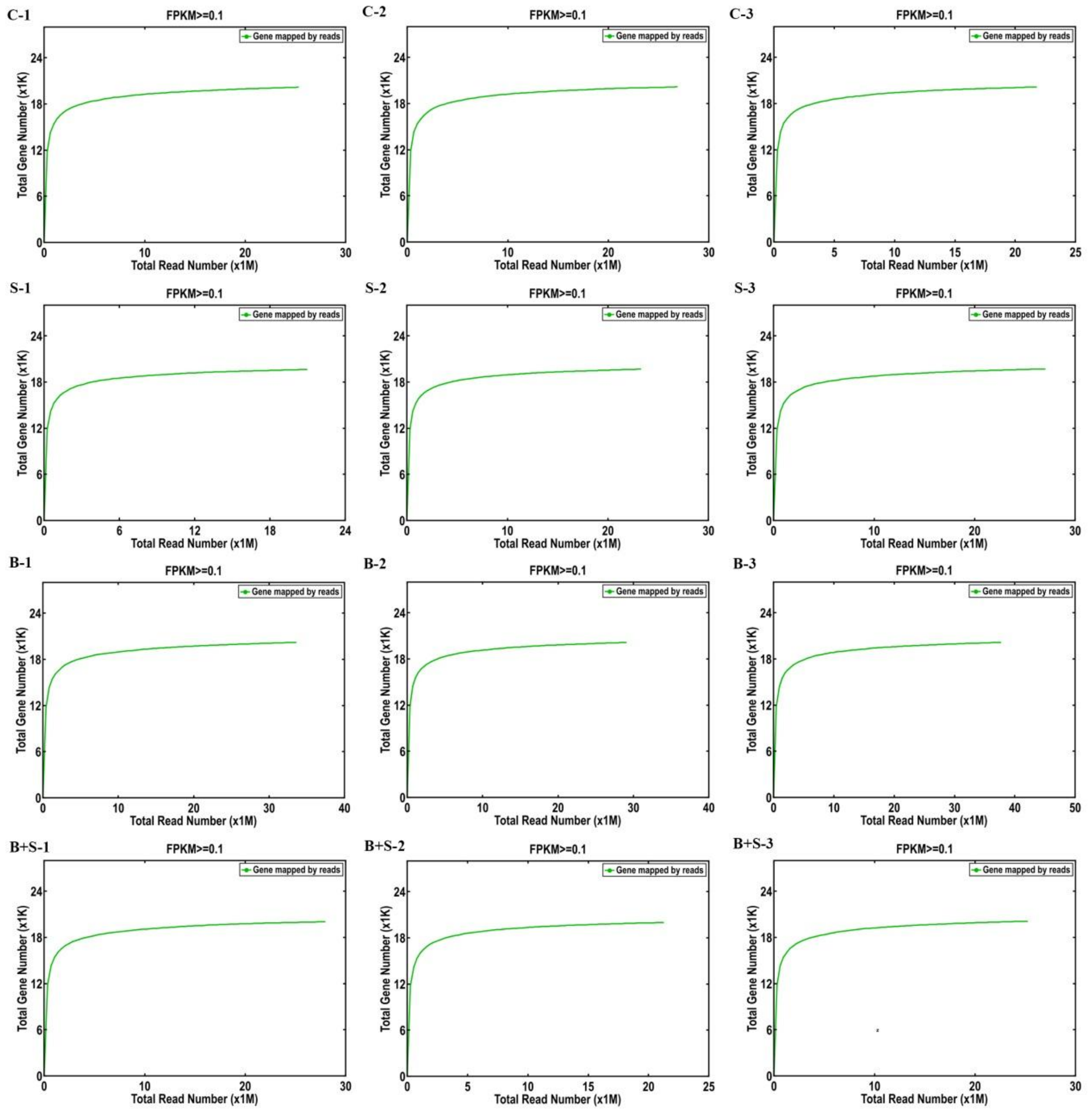
C-1
FPKM>=0.1
Gene mapped by reads
Total Gene Number (x1K)
Total Read Number (x1M)
C-2
FPKM>=0.1
Gene mapped by reads
Total Gene Number (x1K)
Total Read Number (x1M)
C-3
FPKM>=0.1
Gene mapped by reads
Total Gene Number (x1K)
Total Read Number (x1M)
S-1
FPKM>=0.1
Gene mapped by reads
Total Gene Number (x1K)
Total Read Number (x1M)
S-2
FPKM>=0.1
Gene mapped by reads
Total Gene Number (x1K)
Total Read Number (x1M)
S-3
FPKM>=0.1
Gene mapped by reads
Total Gene Number (x1K)
Total Read Number (x1M)
B-1
FPKM>=0.1
Gene mapped by reads
Total Gene Number (x1K)
Total Read Number (x1M)
B-2
FPKM>=0.1
Gene mapped by reads
Total Gene Number (x1K)
Total Read Number (x1M)
B-3
FPKM>=0.1
Gene mapped by reads
Total Gene Number (x1K)
Total Read Number (x1M)
B+S-1
FPKM>=0.1
Gene mapped by reads
Total Gene Number (x1K)
Total Read Number (x1M)
B+S-2
FPKM>=0.1
Gene mapped by reads
Total Gene Number (x1K)
Total Read Number (x1M)
B+S-3
FPKM>=0.1
Gene mapped by reads
Total Gene Number (x1K)
Total Read Number (x1M)

Figure S4

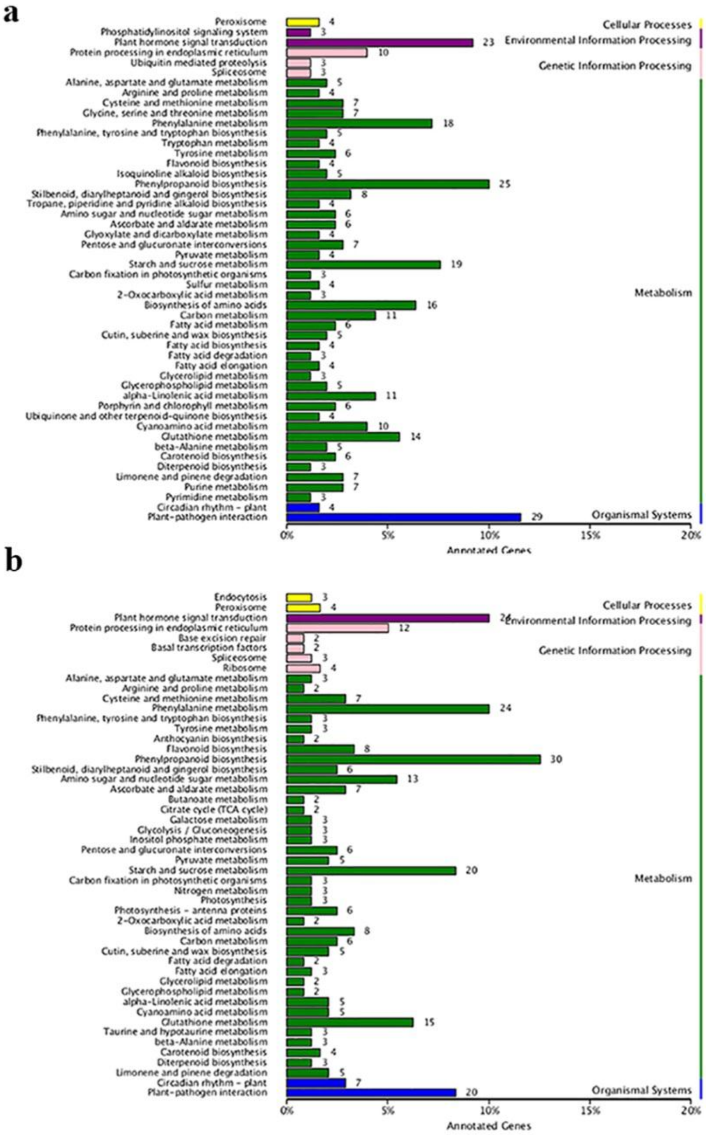

Figure S5

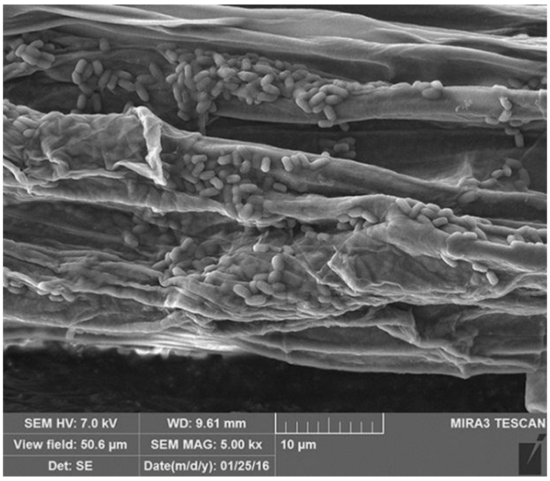

Supplementary Tables

Supplementary Table S1 Reads sequenced under different experimental conditions. (C-1, C-2, and C-3) only water. (S-1, S-2, and S-3) only 100 mM NaCl. (B-1, B-2, and B-3) only FZB42 inoculation. (B+S-1, B+S-2, and B+S-3) 100 mM NaCl + FZB42 inoculation.

Samples	Raw reads (pair-end Reads)	Clean reads (pair-end Reads)	Total mapped reads	Uniq Mapped Reads	Multiple Map Reads	Reads Map to '+'	Reads Map to '-'	GC Content	%≥Q30
C1	25,707,626	25,287,096	42,447,477	41,335,004	1,112,473	21,022,893	21,020,847	45.72%	89.36%
C2	27,198,683	26,814,983	44,812,496	43,409,055	1,403,441	22,138,585	22,134,569	45.76%	89.08%
C3	22,059,945	21,734,952	36,355,044	35,041,247	1,313,797	17,925,179	17,922,841	45.76%	88.88%
S1	21,314,674	20,918,631	35,077,565	33,696,019	1,381,546	17,302,547	17,294,776	45.72%	89.15%
S2	23,654,174	23,225,094	39,141,609	36,871,163	2,270,446	19,142,636	19,136,244	45.94%	89.56%
S3	27,475,956	27,006,276	45,644,056	44,073,510	1,570,546	22,538,308	22,535,804	45.56%	89.41%
B1	34,047,900	33,542,161	57,164,061	52,881,981	4,282,080	28,032,347	28,021,499	46.36%	89.98%
B2	29,522,162	29,033,542	48,892,132	45,346,061	3,546,071	23,742,796	23,738,531	46.34%	89.46%
B3	39,964,370	37,652,903	63,683,484	58,197,691	5,485,793	30,688,191	30,665,743	46.49%	89.95%
B+S-1	28,988,104	27,927,094	47,184,364	43,282,466	3,901,898	22,901,473	22,888,013	46.50%	89.50%
B+S-2	25,589,116	21,218,761	35,203,720	32,263,518	2,940,202	17,064,595	17,051,535	46.25%	88.98%
B+S-3	26,561,635	25,206,561	42,461,178	38,930,904	3,530,274	20,581,178	20,565,973	46.32%	89.37%

Supplementary Table S3 Significantly enriched terms in FZB42-inoculated versus non-inoculated treatments at 0 and 100 mM NaCl (Corrected P-value < 0.05)

0 mM NaCl				
#Kegg_pathway	ko_ID	Corrected_P-value	upregulated	downregulated
Plant-pathogen interaction	ko04626	8.41E-07	23	6
Phenylpropanoid biosynthesis	ko00940	4.78E-05	17	8
alpha-Linolenic acid metabolism	ko00592	5.12E-05	7	4
Phenylalanine metabolism	ko00360	0.002487989	12	6
Glutathione metabolism	ko00480	0.033103794	6	8
100 mM NaCl				
#Kegg_pathway	ko_ID	Corrected_P-value	upregulated	downregulated
Plant-pathogen interaction	ko04626	0.023232437	30	0
Phenylpropanoid biosynthesis	ko00940	1.02E-08	23	1
Phenylalanine metabolism	ko00360	1.23E-07	8	0
Flavonoid biosynthesis	ko00941	0.000392944	11	4
Glutathione metabolism	ko00480	0.00495341	19	1
Photosynthesis - antenna proteins	ko00196	0.041948538	6	0

Supplementary Table S7 RT-PCR primer sequences used in this study.

**Gene_ID**	**Gene putative function**	**Primer(Forward 5'-3')**	**Primer(Reverse 5'-3')**
AT5G44420	ethylene- and jasmonate-responsive plant defensin	CAGAAGTTGTGCGAGAAGCC	GTGCTGGGAAGACATAGTTGC
AT3G23250	myb domain protein 15	CGGAGCTAGCAGATTCATCA	CGTCGTGGCTTATGAGTGTC
AT4G26200	1-aminocyclopropane-1-carboxylate synthase 7	AGACACTCACGGCGAAGACT	ACCTGATTCTCAGCGAGACC
AT1G17420	lipoxygenase 3	TGTGCTTACACCTCCAGTCG	GCATGTGTCCGTAACCAGTG
AT4G39210	glucose-1-phosphate adenylyltransferase large subunit 3	CCGGTGTTGCTTACGCTATT	CCATCGCCTCCTCCTAGAAT
AT4G23700	cation/H(+) antiporter 17	CCGGTGTTGCTTACGCTATT	CCATCGCCTCCTCCTAGAAT
AT1G29910	chlorophyll A/B binding protein 3	AGCGATGGAGGACTCGATTA	CCTGCGACTCTGTAGCCTTC
AT2G05070	photosystem II light harvesting complex protein 2.2	CTCTCTGCTTTGTAAACTCGTGA	CCCAAGAAACATCATTAAGTGCC
ATCG00120	ATP synthase CF1 alpha subunit	TCTTCCGTGGCTCAGGTAGT	CCTGTATAAGGCGCGAGGTA
ATCG00580	photosystem II protein V	TGTCTGGAAGCACAGGAGAA	AACCGGTGCTGACGAATAAC
AT2G39800	delta1-pyrroline-5-carboxylate synthase 1	GCTCGCTTAGTTATGACACCTG	ACCATCTGCCACCTCTGTTT
AT2G05100	photosystem II light harvesting complex protein 2.1	CTCTGCTTTGTAAAGTCGTGAATGT	TGCCAAAACAAACTCACCTGC
AT2G34420	photosystem II light harvesting complex protein B1B2	GCGACGGAGGATTGGACTAC	ATCTCCGGCGACTCTGTAGC
AT3G27690	photosystem II light harvesting complex protein 2.3	GCCACTTCAGCAATCCAACA	TTGACGGTACGACGCATGAT
AT5G54270	light-harvesting chlorophyll B-binding protein 3	TGGCTTCCAAGTCATCCTCA	AACTGGATCATCAGCGAGACC
AT4G39770	probable trehalose-phosphate phosphatase H	GCCAACCAGCTACAGAGTTCC	GCGTCGGAAGTGAACAGAGA
AT1G64170	cation/H(+) antiporter 16	TCTCGGCGACAACAATTCTC	CGGATGTTCAGCCATTCGTA
AT1G14540	peroxidase 4	TCCGATCTTCAATCCGAACC	AGTTGCCACGAGCATGACC
AT1G70290	putative alpha,alpha-trehalose-phosphate synthase	AGCGCGGTCACATAGGTCTT	TCTTCGCCGCAGTAGAATCA
AT3G14440	9-cis-epoxycarotenoid dioxygenase NCED3	CACGACGAGAAGACATGGAA	TCCGATGAATGTACCGTGAA
AT4G13250	probable chlorophyll(ide) b reductase NYC1	CACAACCGAACCAATCACAC	GTGGATCGATGAACGTGTTG
AT1G78090	trehalose-6-phosphate phosphatase	CACTTCCTGGCTAAGGAGAGG	CATCTCCATCGCGTCTGTTA
AT4G36110	SAUR-like auxin-responsive protein	GCGTTGTCTCAAGCAGCATC	CTTCGGTGTTGACCGACGTA

AT5G23370	putative GEM-like protein 8	TCTTCAAGGCGTACCAATGC	AGGAGAAGCCACCTTGATCG
AT3G24500	multiprotein-bridging factor 1c	GGTTCCGGTGATTAACACGA	CGCTTGTGACATCTTCTTCTCC
AT3G03480	acetyl CoA:(Z)-3-hexen-1-ol acetyltransferase	TGAGCTGGAAGAGGCTGATG	TGACCTGGACGAGAAGCAGA
AT4G08390	L-ascorbate peroxidase S	CACCAGGAGGACAGTCATGG	TCGAAGATGGCAGCATCAGT
AT1G05560	UDP-glucosyltransferase 75B1	GAACGCGAAGCTACTGGAAGA	CCACCGACTTCTCCTCCATC
AT5G51720	CDGSH iron-sulfur domain-containing protein NEET	GCTATCATCGCAAGCACGTT	TGCTCTCACCACCACCATTC
AT4G32810	carotenoid cleavage dioxygenase 8	TGGAACATTGGAGACCACGA	AAGCGTCGGATTCAAGGAGA
AT3G12580	heat shock protein 70-4	TCAGCGTCAAGCGACTAAGG	CAACACTCGACGCCTTCTTG
AT3G04800	translocase inner membrane subunit 23-3	GAACTCCGGTGGACTCGTTG	TGTCAACGAACCGTCGTCTC
AT1G64900	cytochrome P450 89A2	GTCGCCAACATGGTCAGAGA	GCAAGAGCCTTAAGCGGATG
AT3G44990	xyloglucan endotransglucosylase/hydrolase	CACCAACGTAGAGAGCAAGACG	GCCTGGTTGGAGCTTAATGG
AT1G10070	branched-chain-amino-acid aminotransferase 2	TGGACAGGCGATATACGAAGG	AACCGAAGGAGAAGGCATGA
AT3G18780	ACT2	AGCAGAGCGGGAAATTGTAA	TTCTCGATGGAAGAGCTGGT
AT2G44065	ribosomal protein L2 family protein	GGGACACCAAGTGGATCAAT	TGGGCCTTATCCCTAACCA

Supplementary Table S2 Specifical and common DEGs in FZB42-inoculated versus non-inoculated plants at 0 and 100 mM NaCl.

Supplementary Table S4 DEGs involved in different metabolic processes at 0 and 100 mM NaCl, respectively, by MapMan analysis.

Supplementary Table S5 Overview of cell functions related to DEGs in FZB42-inoculated versus non-inoculated plants at 0 and 100 mM NaCl, respectively, according to Mapman.

Supplementary Table S6 DEGs of FZB42 inoculated versus FZB42 non-inoculated treatments under non-stress and stress conditions mentioned in results.
